# Geometrical Evolution Pattern and Spectroscopic Properties of Terbium-Doped Germanium Anionic TbGe*_n_* (*n* = 6–17) Nanoclusters: From Tb-Lined to Tb-Encapsulated Structures

**DOI:** 10.3390/molecules30092066

**Published:** 2025-05-06

**Authors:** Chenliang Hao, Jucai Yang

**Affiliations:** 1 Inner Mongolia Key Laboratory of Theoretical and Computational Chemistry Simulation, School of Chemical Engineering, Inner Mongolia University of Technology, Hohhot 010051, China; 2 School of Resources and Environmental Engineering, Inner Mongolia University of Technology, Hohhot 010051, China

**Keywords:** ground-state structure of anionic germanium clusters doped with terbium, structural evolution patterns, magnetism, simulated photoelectron spectroscopy, UV–vis spectra

## Abstract

Developing advanced materials with enhanced performance through the doping of nanoclusters is a promising strategy. However, there remains an insufficient understanding of the specific effects induced by such doped nanoclusters, particularly regarding the structural evolution pattern after doping with rare-earth elements and their impact on performance. To solve this problem, we used first-principles calculation to study the structural evolution pattern and spectroscopic properties of anionic TbGe*_n_* (*n* = 6–17) nanoclusters through the ABCluster global search technique coupled with the mPW2PLYP double-hybrid density functional theory. The results revealed that the geometrical evolution pattern is from the typical Tb-linked structures (for *n* = 10–13, in which Tb acts as a linker connecting two germanium sub-clusters) to Tb-centered cage configurations (for *n* = 14–17). The simulated photoelectron spectroscopy of anionic TbGe_16_ agrees well with its experimental counterpart. Furthermore, we calculated properties such as infrared spectroscopy, Raman spectroscopy, ultraviolet–visible (UV–vis) spectra, magnetism, charge transfer, the HOMO-LUMO gap, and relative stability. The results suggest that TbGe_12_^−^ and TbGe_16_^−^ clusters, with their remarkable stability and tunable photothermal properties, can serve as ideal building blocks for developing novel functional nanomaterials. These clusters demonstrate promising applications in solar photothermal conversion, photoelectric conversion, and infrared imaging technologies through their distinct one- and three-dimensional architectures, respectively.

## 1. Introduction

Doping nanoclusters is one of the promising strategies in materials science for developing advanced materials with enhanced performance, particularly for incorporating rare-earth atoms due to the fact that rare-earth elements possess unfilled 4*f* electrons, which endow them with diverse properties such as excellent magnetic, optical, electronic and catalytic capabilities. Germanium, as a key semiconductor material, is widely used in electronics and optics owing to its high electron mobility, infrared transparency, and high refractive index. Its unique properties make it particularly irreplaceable in infrared technology and specialized electronic devices. As fundamental building blocks of nanomaterials, clusters provide a unique platform for studying the evolution of structural and physical properties across different scales. Consequently, researchers have conducted extensive theoretical [1,2,3,4,5,6,7,8,9,10] and experimental [11,12,13,14,15,16] investigations on both pure germanium clusters and transition-metal-doped germanium clusters.

Doping with a single rare-earth atom (REA) provides distinctive advantages in terms of controllability and directional design for optimizing the physicochemical properties of clusters [16,17,18,19,20,21,22,23,24,25,26,27,28,29]. In terms of experiments, Atobe et al. [16] detected the electron binding energy and stability of TMGe*_n_* (TM = Sc, Y, Lu, Tb, *n* = 8–20) by using anion photoelectron spectroscopy (PES) and the adsorption reactivity of the transition metals to H_2_O. They found that the larger cavity of a Ge_16_ can result in the metal encapsulation configuration of Tb@Ge_16_^−^, Y@Ge_16_^−^, and Lu@Ge_16_^−^ nanoclusters. Stimulated by the experimental observations, some theoretical simulation calculations by means of single- or double-hybrid density functional theory were performed for REA-doped Ge*_n_* clusters such as ScGe*_n_* (*n* = 6–17) [6,17,18], YGe*_n_* (*n* = 6–20) [19], LuGe*_n_* (*n* = 5–17) [20,21,22,23], and their anionic clusters. In addition, Li et al. [24] systematically studied the structural evolution, relative stability, and electron binding energy of LaGe*_n_*⁻ (*n* = 3–14) clusters through single-hybrid density functional theory. They found that the global minimum (GM) of LaGe_11_^−^ and LaGe_14_^−^ clusters is linked structures analogous to their Sc-, Y-, and Lu-doped counterparts [17,18,19,20]. Trivedi et al. [25] investigated the stability and magnetism of EuGe*_n_* (*n* = 1–20) clusters under the framework of single-hybrid density functional theory and reported that the ground-state structures were Eu-encapsulated structures in Ge*_n_* cages with *n* = 14–20. Recently, our group investigated the structural growth patterns and electronic properties of REAs (REA = Sc, Y, Lu, Ce, Eu, Gd) incorporated into anionic Ge*_n_*^−^ (*n ≤* 20) nanoclusters by means of double-hybrid density functional theory. The results revealed that their growth patterns transition from REA-linked to REA-encapsulated configurations as the cluster size *n* increases [18,19,20,26,28,29]. Although all REA-doped clusters adopt linked structures, their ground-state geometries vary significantly for a given cluster size, as demonstrated in this study. In this work, we selected terbium-doped anionic germanium clusters as the research focus, not only to extend our previous work but also to leverage terbium’s unique electronic structure, which enables its application in functional materials such as sensitizing agents, magnetic distortion materials, and magneto-optical storage devices. Incorporating a Tb atom into an anionic Ge*_n_* cluster not only enhances its stability but also introduces novel magnetic and photoelectric activity, positioning it as a promising multifunctional fundamental unit for downsized semiconductor materials. The aim of this work is to elucidate the geometric stability and growth pattern of the nanocluster GM structures, quantify thermochemical parameters, simulate spectral properties, and provide critical insights for advancing theoretical and experimental studies on terbium-doped (or other metal-doped) semiconductor nanoclusters.

## 2. Computational Details

The detailed calculation method has been described elsewhere [18,19,20,26,27,28], and here is a brief description. Three techniques were applied to search for the initial structures of anionic TbGe*_n_* (*n* = 6–17) nanoclusters. Firstly, with the ABCluster (artificial bee colony (ABC) for cluster) global search technique [30,31,32] coupled with the Gaussian 09 software package [33], larger than 300 geometries for each anionic TbGe*_n_* clusters were optimized by means of single-hybrid TPSSh density functional theory [34] with the pseudopotential ECP54MWB basis set [35,36] for Tb atoms and the ECP28MWB basis set for Ge atoms [37]. Secondly, “substitutional structure” tactics were employed, where a Tb atom substitutes for a Ge atom in the GM configuration of neutral Ge*_n_*_+1_ and/or anionic Ge*_n_*_+1_^−^ clusters. Thirdly, GM spatial structures already presented in earlier articles [16,17,18,19,20,21,22,23,24,25,26,27,28,29] were adopted. The gained low-lying geometries were reoptimized through the single-hybrid density functional TPSSh coupled with the def2-TZVP basis set [38] for Tb atoms and the cc-pVTZ-PP basis set [39,40] for Ge atoms. All TbGe*_n_*^−^ (*n* = 6–17) stationary point geometries were interrogated by the prediction of their harmonic vibrational frequencies at the same level. After settlement of the primary isomer optimization through TPSSh, we picked the low-lying structural candidates and reoptimized them through a double-hybrid functional mPW2PLYP [41] with the def2-TZVP basis set [38] for Tb atoms and the cc-pVTZ-PP basis set [39,40] for Ge atoms (the frequency calculation was not executed at the mPW2PLYP level of theory). At length, to further refine the energy, single-point energy calculation was performed by means of the mPW2PLYP functional with the def2-TZVP basis set [38] for Tb atoms and the aug-cc-pVTZ basis set [42] for Ge atoms (denoted as LARGE-BS). The PES spectra of TbGe*_n_*^−^ (*n* = 6–17) clusters were simulated based on the OVGF (outer-valence Green function) scheme [43] in combination with the def2-TZVP basis set [38] for Tb atoms and the cc-pVTZ basis set [42] for Ge atoms and compared with the effective experimental data. To comprehend in depth the interaction between Tb atoms and germanium clusters, natural population analyses (NPAs) were also performed by the mPW2PLYP functional with LARGE-BS. The ultraviolet–visible (UV–vis) spectra were simulated through time-dependent density functional theory with the PBE0 functional [44,45] and LARGE-BS. The visualization results were achieved through Multifwn (Ver. 3.8) [46,47] and VMD 1.9.3 software [48]. Furthermore, the spin multiplicities of septet and nonet states were considered for anionic TbGe*_n_*^−^ (*n* = 6–17) nanoclusters. The reliability of our calculation has been tested previously [18,19,20,49,50]. All calculations were performed using the Gaussian 09 software package [33].

## 3. Result and Discussion

### 3.1. GM Configurations of Anionic TbGe_n_ (n = 6–17) Nanoclusters

**Figure 1:**
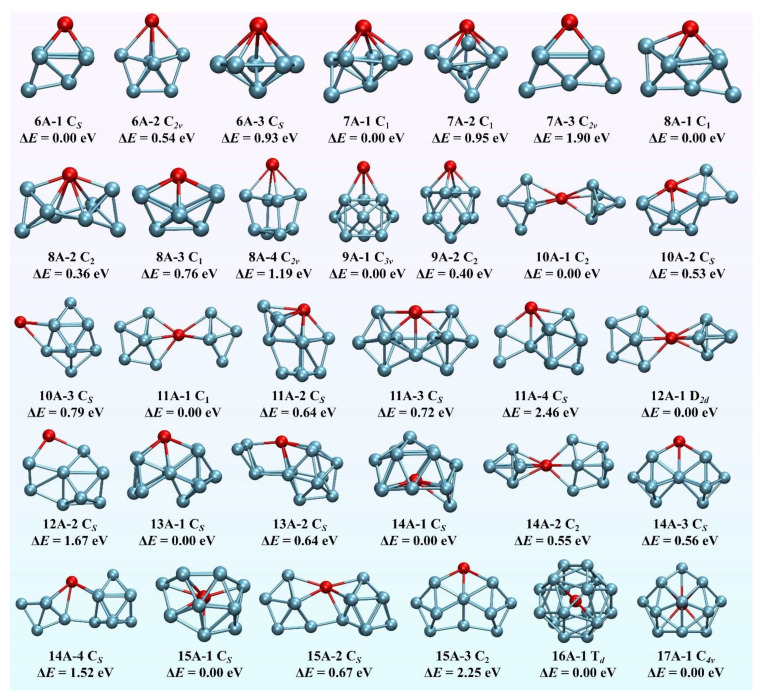
The GM structures and their isomers of TbGe*_n_*^−^ (*n* = 6–17) obtained at the mPW2PLYP level. The red and cyan (color online) stand for Tb and Ge atoms, respectively.

The GM configurations and selected low-lying isomers for anionic TbGe*_n_* (*n* = 6–17) nanoclusters obtained at the mPW2PLYP level of theory are shown in Figure 1. Each nanocluster is named *n*A-*x*, in which *n* is number of Ge atoms, A is the anion, and *x* is the number of isomers. The electronic states for anionic TbGe*_n_* (*n* = 6–17) were all evaluated to septet states. For *n* = 6, three isomers are presented. The *C_s_*-symmetry **6A-1** is predicted to be the GM configuration, which differs from that of REAGe_6_^−^ (REA = Sc, Y, Lu, Ce, Gd) species [18,19,20,26,27]. Their GM structures are analogous to **6A-3**. It is also different than that of the SmGe_6_^−^ cluster, which is analogous to the **6A-2** isomer. The **6A-2** and **6A-3** geometries are less stable in energy than the geometry of the **6A-1** GM structure by 0.54 and 0.93 eV, respectively. For *n* = 7, three geometries are reported. The *C*_1_-symmetry **7A-1** is evaluated to be the GM structure. It differs from those of the REAGe_6_^−^ (REA = Y, Lu, Gd) species [19,20,27]. Their GM geometries are analogous to the **7A-3** isomer. The **7A-2** and **7A-3** isomers are less stable in energy than the isomer of **7A-1** by 0.95 and 1.90 eV, respectively. For *n* = 8, four isomers are displayed. The GM configuration is calculated to be *C*_1_-symmetry **8A-1**, analogous to the GM of anionic GdGe_8_ [27]. The *C*_2_-symmetry **8A-2** is less stable in energy than that of **8A-1** by 0.36 eV. The isomer **8A-3** has approximately *C_s_*-symmetry, but there is no symmetry constraint during the calculation. It is analogous to the GM structure of the ScGe_8_⁻ and LuGe_8_^−^ clusters [18,20]. The *C*_2*v*_-symmetry **8A-4** isomer is analogous to the GM geometry of SmGe_8_^−^ and EuGe_8_^−^ clusters [28,29]. Energetically, the **8A-3** and **8A-4** isomers are higher than the isomer of **8A-1** by 0.76 and 1.19 eV, respectively. For *n* = 9, two isomers are presented. The *C*_3*v*_-symmetry **9A-1** is predicted to be the GM configuration. It differs from that of REAGe_9_^−^ (REA = Sc, Y, Lu, Ce, Gd, Sm, Eu) [18,19,20,26,27,28,29], of which the GM structures are analogous to that of the **9A-2** isomer. Energetically, **9A-2** is higher than **9A-1** by 0.40 eV. For *n* = 10, three isomers are shown. The GM configuration is calculated to be *C*_2_-symmetry **10A-1**, analogous to the GM of anionic REAGe_10_ (REA = Sc, Y, Lu, Ce, Gd) [18,19,20,26,27]. It is a linked structure, in which the Tb atom links two Ge_5_ trigonal bipyramids. The *C_s_*-symmetry **10A-2** isomer is less stable in energy than that of **10A-1** by 0.53 eV. The *C_s_*-symmetry **10A-3** isomer is analogous to the GM structure of the SmGe_10_^−^ and EuGe_10_^−^ clusters. Energetically, it is predicted to be 0.79 eV above the **10A-1** structure. For *n* = 11, four isomers are presented. The *C*_1_-symmetry **11A-1** is predicted to be the GM structure. It belongs to the linked structure, in which the Tb atom links two subgroups of the Ge_5_ trigonal bipyramid and Ge_6_ capped trigonal bipyramid. It is analogous to the GM configurations of anionic REAGe_11_ (REA = Sc, Y, Lu, Ce, Gd) [18,19,20,26,27]. It is different from the GM structures of the SmGe_11_^−^ and EuGe_11_^−^ clusters because their GM belongs to the adsorption structure [28,29]. The **11A-2**, **11A-3**, and **11A-4** isomers in the *C_s_*-symmetry are calculated to 0.64, 0.72, and 2.46 eV above GM **11A-1**, respectively. For *n* = 12, two isomers are displayed. The GM structure is predicted to be *D*_2*d*_-symmetry-linked geometry **12A-1**, in which the Tb atom links two Ge_6_ capped trigonal bipyramids. It is analogous to the GM structure of the YGe_12_^−^, LuGe_12_^−^ and GdGe_12_^−^ clusters [19,20,27]. The *C_s_*-symmetry **12A-2** is analogous to the GM geometry of the SmGe_12_^−^ and EuGe_12_^−^ clusters [28,29]. Energetically, it is evaluated to be 1.67 eV above the **12A1** GM. For *n* = 13, two isomers are presented. The *C_s_*-symmetry **13A-1** is predicted to be the GM structure. It can be seen as a linked structure, in which the Tb atom links two subgroups of the Ge_5_ approximate planar five-membered ring and the Ge_8_ square antiprism. It differs from those of the YGe_13_^−^, LuGe_13_^−^, GdGe_13_^−^, SmGe_13_^−^, and EuGe_13_^−^ clusters [19,20,27,28,29], of which the GM structure is analogous to the **13A-2** isomers. The **13A-2** isomer is also a linked structure, in which the Tb atom links two subgroups of the Ge_4_ approximate planar four-membered ring and the Ge_9_ tricapped trigonal prism (TTP). Energetically, it is less stable than that of **13A-1** by 1.10 eV. For *n* = 14, four isomers are shown. The *C_s_*-symmetry **14A-1**, an incomplete cage structure, is predicted to be the GM configuration. It is more stable in energy than the linked structures of **14A-2** in the C_2_-symmetry and **14A-4** in the *C_s_*-symmetry by 0.55 and 1.52 eV, respectively. The **14A-3** structure belongs to the substitutional structure. It is predicted to 0.56 eV above the **14A-1** cage-like structure. For *n* = 15, three isomers are reported. The GM structure is predicted to be Tb-centered in the Ge-cage configuration (*C_s_*-symmetry **15A-1**), of which the cage can be viewed as being derived from fullerene of Ge_16_ by removing a Ge atom analogous to that of the GdGe_15_^−^ cluster [27]. The *C_s_*-symmetry **15A-2** belongs to the linked structure, in which the Tb links two subgroups of a Ge_6_ capped trigonal bipyramid and Ge_9_ TTP. The *C*_2_-symmetry **15A-3** belongs to the substitutional structure, in which the Tb atom substitutes for a Ge atom in the GM configuration of neutral Ge_16_ clusters [1,2,3]. Energetically, the **15A-2** and **15A-3** isomers are calculated to 0.67 and 2.25 eV above that of the **15A-1** structure, respectively. For *n* = 16, its GM structure (*T_d_*-symmetry **16A-1**) is predicted to be Tb-centered in the Frank–Kasper (FK) cage of Ge_16_. This result is consistent with the experimental observations [16]. For *n* = 17, the GM structure is evaluated to be the Tb-centered Ge-cage configuration with *C*_4*v*_-symmetry (**17A-1**).

After discussing the GM configuration, we concentrate on the equilibrium geometrical evolution of the anionic TbGe*_n_* (*n* = 6–17) nanocluster at present. According to the structural traits of the determined GM configuration, the equilibrium geometrical evolution patterns are inclined to the Tb-linked configuration, in which the Tb atom links two Ge sub-clusters beginning from *n* = 10, and the Tb-centered Ge cage polyhedron is preferred when *n* reaches 14. In fact, since Kumar and coworkers first reported the formation of linked clusters and endohedral cages in Y-doped anionic silicon clusters [51], it has been found that REA-doped anionic semiconductor clusters do exhibit a transition pattern from the linked structure to the cage configuration. Different rare-earth dopants and semiconductor clusters will form different linked structures and/or endohedral cages. This is because the GM configuration form depends on the diameter and electronic structure of dopants and host atoms. For example, the atomic radii of Sm and Eu are larger than that of Tb. Therefore, the minimum cluster size required to form a cage-like structure is *n* = 14 for Tb-doped anionic Ge nanoclusters (Tb@Ge*_n_*^−^), compared to *n* = 20 for Sm-doped clusters [28]. In contrast, no cage-like structure is observed in the Eu-doped anionic germanium clusters even at *n* = 20 [29], and their minimum cage-forming size is approximately *n* = 26. The case of *n* = 9 serves as the most illustrative example of how electronic structures influence GM configurations. Ce and Gd exhibit an oxidation state of +3 in the CeGe_9_^−^ and GdGe_9_^−^ clusters [26,27], originating from their 5*d*^1^6*s*^2^ electron configuration, whereas Tb adopts the +4 oxidation state in TbGe_9_^−^ derived from its 4*f*^2^6*s*^2^ configuration (see below). These distinct electronic structures lead to different structures: the TbGe_9_^−^ exhibits *C*_3*v*_-symmetry geometry, while the CeGe_9_^−^ and GdGe_9_^−^ clusters adopt the *C*_4*v*_-symmetry configuration.

### 3.2. The Charge Transfer and Magnetic Moment of TbGe_n_^−^ (n = 6–17) Nanoclusters

To better understand the interaction between Tb atoms and Ge clusters or the influence of electronic structure on structure and property, we then conducted a natural population analysis (NPA) of the anionic TbGe*_n_*^−^ (*n* = 6–17) clusters. The data including charge, valence electron configuration, and magnetic moments of each orbital and the total configuration of anionic TbGe*_n_*^−^ (*n* = 6–17) clusters are listed in Table 1. It can be seen from Table 1 that (i) the valence configuration of Tb in the anionic TbGe*_n_* nanoclusters is 6*s*^0.66–1.28^4*f*^6.99–7.38^5*d*^1.73–5.28^6*p*^0.29–2.00^7*s*^0.10–0.41^. This means that the 4*f* electrons of the Tb ([Xe]4*f*^9^6*s*^2^) atom are involved in bonding. The way to involve in bonding is initially transferring 4*f* electrons to the 5*d* and/or 6*p* orbital, and then the 5*d* and/or 6*p* orbital participates in bonding with valence electrons of the Ge*_n_* nanocluster. To be precise, in addition to the 4*f* electron transition to the 5*d* orbital, 6*s* electrons also transfer to the 5*d* and/or 6*p* orbitals, leading to hybridization between the 6*s* and 5*d* (and/or 6*p*) orbitals. (ii) For the *n* = 6–8,11–13,14,16,17 clusters, one 4*f* orbital electron transition to that of 5*d* reveals that the oxidation state of the Tb atom in these cluster is three. For clusters of *n* = 9,10,15, their oxidation numbers are four because there are two 4*f* orbital electrons that transfer to 5d orbitals. (iii) For clusters with *n* = 6–8,10,11,13, the charge of Tb in TbGe*_n_*^−^ is within +0.07 to +0.39 a.u. These results imply that terbium serves as an electron donor, thereby suggesting that ionic bonding dominates between Tb and the germanium network. For clusters of *n* = 9,12,14–17, the atomic charge of Tb ranges from −0.27 to −4.76 a.u., demonstrating its role as an electron acceptor. Furthermore, the bonding between Tb and the germanium cluster framework is predominantly metallic in nature. (iv) The total magnetic moments of TbGe*_n_*^−^ (*n* = 6–17) were kept at a fixed value of 6 μ*_B_* and were contributed by the Tb atom.

### 3.3. The Evaluated Photoelectron Spectroscopy of TbGe_n_^−^ (n = 6–17)

**Figure 2:**
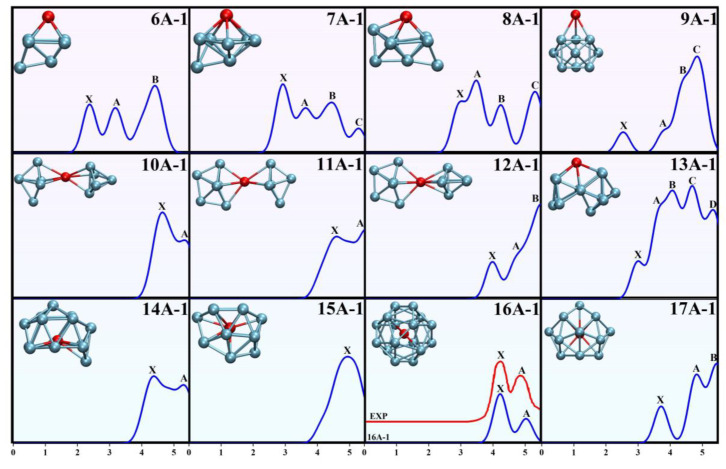
Simulated and experimental PES spectra of the anionic TbGe*_n_*^−^ (*n* = 6–17) clusters (in eV). The blue and red curves (color online) represent the simulated and experimental PES curves [16], respectively.

Owing to the multiple isomers and states of nanoclusters, there are no experimental methods for directly measuring the GM of nanoclusters so far. Photoelectron spectroscopy is highly sensitive to changes in both the electronic and geometric structure of anionic clusters. Therefore, PES is one of the significant techniques for indirectly verifying the spatial geometric shape and electronic structure of nanoclusters. Given the crucial importance of the PES characterization of nanoclusters, we simulated the photoelectron spectra of the clusters. The simulated energy range was set between 0 and 5.5 eV, with a full width at half maximum (FWHM) of 0.50 eV using a Gaussian function. The first simulated peak was defined as X (the vertical detachment energy (VDE)), followed by subsequent peaks labeled as A, B, C, and so on corresponding to ascending energy positions. The results of the simulated spectra are summarized in Figure 2. When *n* = 6, three consecutive peaks can be observed in the spectrum, located at 2.38 eV (X), 3.18 eV (A), and 4.41 eV (B), respectively. When *n* = 7, four identifiable peaks, X, A, B and C, are present at 2.92, 3.63, 4.44, and 5.27 eV, respectively. For *n* = 8, the spectrum exhibits three prominent peaks, A (3.48 eV), B (4.24 eV), and C (5.31 eV), along with a minor shoulder peak X (3.02 eV) preceding peak A in the lower-energy region. When *n* = 9, the PES features a relatively isolated peak X (2.53 eV) and peak C (4.44 eV), accompanied by two shoulder peaks A (3.80 eV) and B (4.44 eV) on the lower-energy side of peak C. The number of peaks in the PES of **7A-1**, **8A-1**, and **9A-1** is the same, but they can be distinguished by differences in peak shape and position. When *n* = 10, the spectrum exhibits a distinct X peak at 4.64 eV, accompanied by peak A at 5.33 eV. For *n* = 11, two weakly resolved peaks, X (4.60 eV) and A (5.49 eV), can be observed within the fitted energy range. When *n* = 12, the PES shows three distinct peaks: an isolated X peak at 3.99 eV, a prominent B peak at 5.49 eV, and peak A (4.77 eV) on the lower-energy shoulder of peak B. Due to the structural similarity of **10A-1**, **11A-1**, and **12A-1**, their photoelectron spectra are quite similar, especially between **10A-1** and **11A-1**, where the peak positions and the number of peaks display minimal differences. Therefore, careful distinction is required in the experimental analyses. When *n* = 13, four distinct peaks, X (3.01 eV), B (4.07 eV), C (4.68 eV), and D (5.33 eV), along with a shoulder peak A (3.63 eV) adjacent to B, are observed in the PES. When *n* = 14, the spectra exhibit two peaks, X (4.38 eV) and A (5.30 eV). For *n* = 15, only a single peak, X (4.94 eV), is observed within the investigated energy range. For TbGe_16_^−^, the simulated PES was compared with the experimental spectrum. The simulated spectrum shows two peaks: X at 4.22 eV and A at 5.03 eV. The experimental PES also detected two peaks, located at 4.23 eV and 4.86 eV [16]. It is worth noting that the simulated PES shows excellent consistency with the experimental PES in terms of peak shape, the number of peaks, and peak positions. When *n* = 17, within the 0–5.5 eV range, the spectrum exhibits an isolated peak X at 3.71 eV, followed by two consecutive peaks, A (4.82 eV) and B (5.44 eV). Overall, the consistency between the theoretical and experimental PES spectra of the TbGe_16_^−^ cluster validates the reliability of our predicted GM structure. The simulated spectra can provide an important theoretical reference and guidance for future related experimental studies.

### 3.4. The Simulated Infrared and Raman Spectra of TbGe_n_^−^ (n = 6–17)

**Figure 3:**
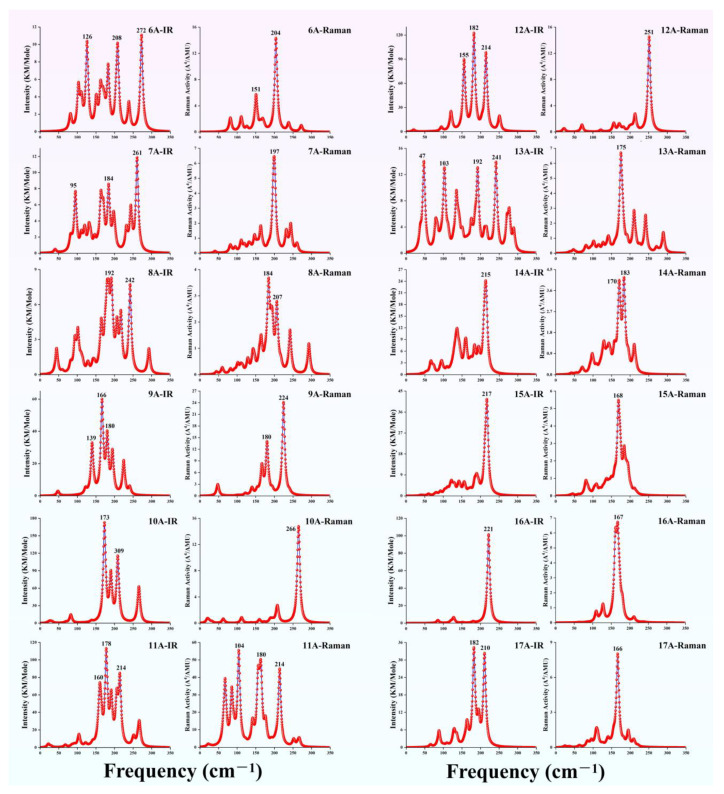
IR and Raman spectra of GM structures for TbGe*_n_*^−^ (*n* = 6–17) clusters.

In addition to PES, infrared (IR) and Raman spectroscopy serve as critical techniques for indirectly validating the GM structures of clusters. By integrating computational simulations with the spectral analysis of IR and Raman data for TbGe*_n_*^−^ clusters (*n* = 6–17), we systematically characterize the vibrational signatures of their GM configurations, thereby establishing a predictive framework for the experimental determination of cluster geometries. All simulations were performed at the TPSSh level, employing the def2-TZVP basis set for Tb atoms and the aug-cc-pVTZ basis set for Ge atoms. The calculated spectra, presented in Figure 3, exhibit no imaginary frequencies, confirming the thermodynamic stability of these configurations. For the TbGe_6_^−^ cluster, the IR spectrum exhibits three prominent peaks accompanied by several minor ones. The most intense vibrational peak at 272 cm^−1^ is attributed to the Ge-Ge stretching vibration, with the Tb atom remaining stationary. The secondary peak at 126 cm^−1^ arises from the scissoring vibration of the Ge-Tb-Ge moiety, while the peak at 208 cm^−1^ corresponds to an in-plane rocking vibrational mode. In the Raman activity spectrum, a dominant peak emerges at 204 cm^−1^, which belongs to the symmetric stretching vibration. For the TbGe_7_^−^, its IR spectrum demonstrates the strongest vibration at 261 cm^−1^, originating from asymmetric stretching vibrations. The second most intense peak at 184 cm^−1^ is associated with in-plane deformation vibrations, whereas the feature peak at 95 cm^−1^ results from coupled scissoring and symmetric stretching motions. The Raman spectrum shows a characteristic intense peak at 197 cm^−1^, which arises from the degeneracy of two distinct stretching vibrational modes. For TbGe_8_^−^, the strongest peak in the IR spectrum corresponds to multiple stretching vibrations at 192 cm^−1^. The second strongest peak, located at 242 cm^−1^, is attributed to the breathing vibration of the trigonal pyramidal Ge_4_ cluster. In the Raman spectrum, the most intense characteristic peak appears at 184 cm^−1^, corresponding to a deformation vibration. For TbGe_9_^−^, three prominent peaks, ranked by intensity, appear at 166 cm^−1^, 180 cm^−1^, and 139 cm^−1^, which are attributed to multiple out-of-plane rocking vibrations, the breathing vibration of the trigonal pyramidal TbGe_3_ unit, and anti-symmetric stretching vibrations, respectively. The most intense characteristic peak in the Raman spectrum is observed at 224 cm^−1^, corresponding to the breathing vibration of the Ge_9_ sub-cluster in a TTP structure, with the Tb atom remaining relatively stationary. The second strongest peak, located at 180 cm^−1^, arises from symmetric stretching vibrations. In the IR spectrum of TbGe_10_^−^, the strongest peak, located at 173 cm^−1^, is caused by anti-symmetric stretching involving two Ge-Tb-Ge units. The second characteristic peak, appearing at 309 cm^−1^, originates from the coupled scissoring vibrations of two Ge_5_ sub-clusters. The Raman spectrum exhibits only one prominent characteristic peak, attributed to the breathing vibrations of the two Ge_5_ sub-clusters, with the Tb atom remaining relatively stationary. For TbGe_11_^−^, the IR spectrum shows that the stronger characteristic peaks are concentrated, with the most intense peak at 178 cm^−1^, resulting from a combination of stretching and scissoring vibrations. The second strongest peak, located at 214 cm^−1^, is attributed to in-plane rocking vibrations of the Ge-Tb-Ge units and symmetric stretching vibrations. In contrast to the IR spectrum, the characteristic peaks in the Raman spectrum are more dispersed, with three strong peaks appearing at 104 cm^−1^, 166 cm^−1^, and 213 cm^−1^, which correspond to in-plane rocking, scissoring, and stretching vibrations, respectively. The IR spectrum of TbGe_12_^−^ exhibits three distinct peaks, with the strongest peaks at 182 cm^−1^ and 214 cm^−1^, both attributed to symmetric stretching vibrations, while the peak at 155 cm^−1^ arises from scissoring vibrations. The Raman spectrum features a single strong characteristic peak at 251 cm^−1^, originating from two degenerate breathing vibrations. In the IR spectrum of TbGe_13_^−^, four peaks of nearly equal intensity are observed, with the peak at 47 cm^−1^ arising from out-of-plane rocking vibrations, the peak at 103 cm^−1^ attributed to double stretching vibrations, and the peaks at 192 cm^−1^ and 241 cm^−1^ originating from symmetric stretching motions involving different atoms. The Raman spectrum exhibits significantly fewer characteristic peaks than the IR spectrum, with only one prominent peak at 175 cm^−1^, which is attributed to symmetric stretching vibrations. The most prominent feature in the infrared spectrum of the cage-like TbGe_14_^−^ cluster is attributed to a dual bending vibration, appearing at 215 cm^−1^. In contrast, the Raman spectrum exhibits two significant peaks: one at 183 cm^−1^ arising from a mixed vibrational mode involving in-plane wagging and stretching motions and another at 170 cm^−1^ due to symmetric deformation vibrations. The IR and Raman spectra of TbGe_15_^−^ both exhibit a single prominent characteristic peak, corresponding to the triply degenerate bending vibration at 217 cm^−1^ and the symmetric deformation vibration at 168 cm^−1^, respectively. Similar to TbGe_15_^−^, the TbGe_16_^−^ cluster with T*_d_* symmetry also displays a dominant strongest peak in both its IR and Raman spectra. These are attributed to the triply degenerate bending vibration at 221 cm^−1^ and the stretching vibration of the Ge_16_ cage at 167 cm^−1^, respectively, where the Tb atom remains stationary. The infrared spectrum is highly active, while the Raman spectrum is inactive due to the structure having a center of symmetry, which follows the centrosymmetric rule. The analysis of the infrared and Raman spectra reveals that before the Tb atom is encapsulated by Ge, both spectra exhibit numerous characteristic peaks, particularly in the IR spectrum. However, upon the formation of a cage-like cluster structure, the number of significant peaks in both vibrational spectra is notably reduced and becomes more distinct. These simulated spectra provide robust evidence to support future qualitative experiments on such clusters. Moreover, since all the observed peaks are located in the far-infrared region, these ground-state clusters hold promising potential for application in the development of infrared sensing devices.

### 3.5. The Relative and Chemical Stabilities of TbGe_n_^−^ (n = 6–17)

The assessment of cluster stability is a critical focus in nanocluster research. In this study, the stability of clusters is investigated by calculating the average binding energy (*ABE*), the second-order energy difference (Δ^2^*E*), and the HOMO-LUMO energy gap (*E*_gap_). The higher the value of the *ABE* is, the more stable the clusters of TbGe*_n_*^−^ (*n* = 6–17) is, and Δ^2^*E* reflects the relative stability between clusters of adjacent sizes in a system. *E*_gap_, to some extent, indicates the photochemical stability of the clusters. The formulas for calculating the three parameters are as follows:

(1) $$ABE({\mathrm{TbGe}}_{n}^{-})=\frac{\left(n-1)E(\mathrm{Ge})+E({\mathrm{Ge}}^{-})+E(\mathrm{Tb})-E({\mathrm{TbGe}}_{n}^{-}\right)}{n+1}$$

(2) $$\Delta^{2}E({\mathrm{TbGe}}_{n}^{-})=E({\mathrm{TbGe}}_{n-1}^{-})+E({\mathrm{TbGe}}_{n+1}^{-})-2E({\mathrm{TbGe}}_{n}^{-})$$

(3) $$E_{gap}=E(\mathrm{LUMO})-E(\mathrm{HOMO})$$

**Figure 4:**
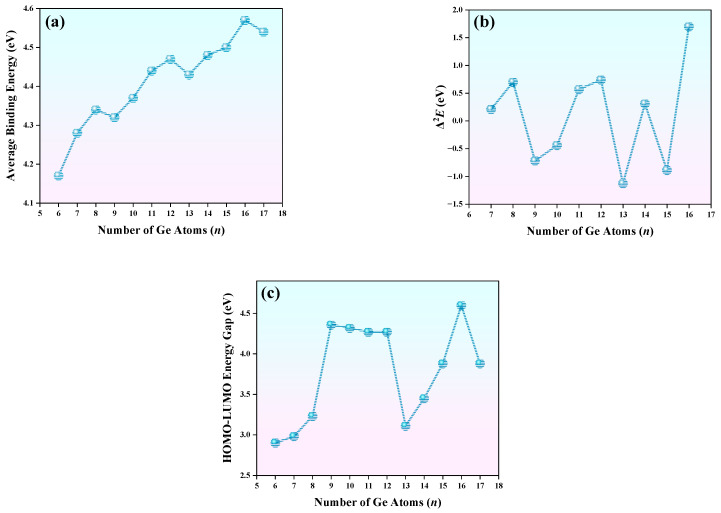
The (**a**) average binding energy (*ABE*, in eV), (**b**) second-order difference energy (Δ^2^*E*, in eV), and (**c**) HOMO-LUMO gap (*E*_gap_, in eV) of the TbGe*_n_*^−^ (*n* = 6–17) cluster.

Figure 4a illustrates the trend of *ABE* variation with cluster size. The *ABE* generally increases as the size of the cluster increases. When the Δ*ABE* increment diminishes, a local maximum point will be formed, and the cluster corresponding to this local maximum point is relatively stable. Distinct stability minima are observed at *n* = 9 and 13, manifested by a slight decrease in *ABE* values. The stability progression reaches its maximum at *n* = 16, where the *ABE* peaks at 4.57 eV within the studied size range. Another intriguing feature observed in the *ABE* curve suggests potential periodicity in stability patterns, where clusters with sizes corresponding to multiples of four (*n* = 4k, k is a natural number) exhibit enhanced structural stability. Figure 4b shows the Δ^2^*E* analysis. It can be clearly seen from Figure 4b that clusters *n* = 8, 12, 14, and 16 are locally stable. These observational results indicate that clusters *n* = 8, 12, and 16 will be highly prominent in their mass spectrometry distributions. Figure 4c presents the *E*_gap_ values for clusters of different sizes. As evident from Figure 4c, the TbGe*_n_*^−^ (*n* = 6–17) nanoclusters with *n* = 16 exhibit the largest *E_gap_* (4.60 eV), followed by clusters *n* = 9–12 (their Egap values differ from each other, varying within the range of 4.27–4.36 eV), while cluster *n* = 6 demonstrates the smallest *E_gap_* (2.90 eV). These results correlate with chemical stability trends, where the *n* = 16 cluster displays optimal stability, follow by clusters with *n* = 9–12. The *E*_gap_ calculated using the pure density functional theory is smaller than that calculated by the hybrid DFT. This occurs because the Kohn–Sham molecular orbital approximations predict similar energy upshifts for both the HOMO and LUMO, whereas the Hartree–Fock (HF) method shifts the LUMO to significantly higher energy levels compared to the HOMO [52]. For example, for the YGe*_n_*^−^ (*n* = 6–20) cluster, the *E*_gap_ predicted by mPW2PLYP is larger than that of the PEB by 2.33 eV [19]. In summary, clusters with *n* = 12 and 16 exhibit not only superior thermal stability but also optimal chemical stability, making them prime candidates as building blocks for evolving into novel functional nanomaterials with tunable one- and three-dimensional architectures.

### 3.6. UV–Vis Spectra of the TbGe_12_^−^ and TbGe_16_^−^ Clusters

**Figure 5:**
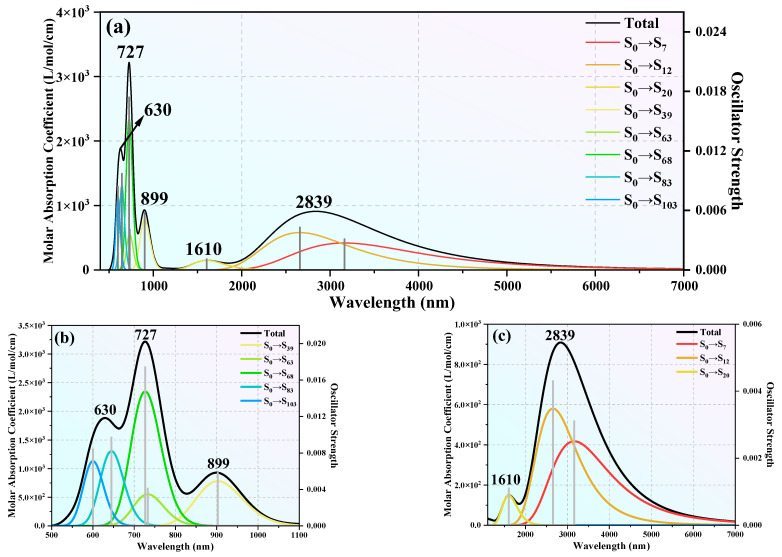
The (**a**) total UV–vis spectrum and localized spectra from (**b**) 400–1100 nm and (**c**) 1100–7000 nm of TbGe_12_^−^.

**Figure 6:**
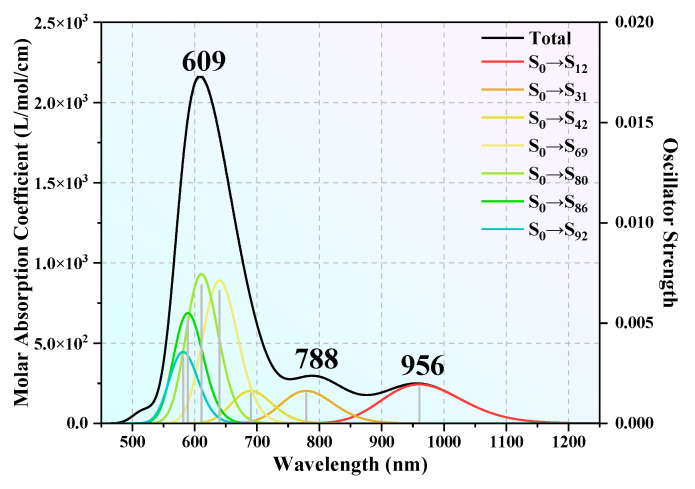
The UV–vis spectrum of the TbGe_16_^−^ cluster.

As discussed above, clusters with *n* = 12 and 16 not only have superior thermal and chemical stability but also represent prototypical examples of Tb-linked and Tb-centered caged architectures, respectively. Consequently, these two clusters were selected for further investigation of their ultraviolet absorption (UV–vis) spectra. Time-dependent density functional theory (TD-DFT) calculations were performed, with 120 excited states computed for each cluster to ensure a comprehensive description of their optical properties. The maximum half-peak width of the spectrum was set to 0.25 eV. The simulated UV–vis spectra of the TbGe_12_^−^ and TbGe_16_^−^ clusters are shown in Figure 4 and Figure 5, respectively. Figure 5a displays the total UV–vis spectrum of TbGe_12_^−^ across a broad wavelength range of 400–7000 nm. For enhanced clarity, the spectrum is subdivided into two regions for detailed analysis: 400–1100 nm and 1100–7000 nm. As shown in Figure 5a, TbGe_12_^−^ exhibits five distinct absorption peaks spanning the visible (380–780 nm), near-infrared (780–2500 nm), and mid-infrared (2500–25,000 nm) regions. The two most intense absorption peaks occur in the visible region at 727 nm and 630 nm. Two weaker peaks are observed in the near-infrared region at 899 nm and 1610 nm, while the mid-infrared region features a broad absorption peak with maximum intensity at 2839 nm. The strongest absorption peak at 727 nm arises predominantly from the S_0_→S_68_ (72.91%) and S_0_→S_63_ (16.91%) transitions, where “S” denotes a septet. The secondary peak at 630 nm is primarily attributed to S_0_→S_83_ (60.89%) and S_0_→S_103_ (30.44%) transitions. The third strongest peak at 2839 nm results from contributions by S_0_→S_12_ (59.95%) and S_0_→S_7_ (40.4%). The minor peak at 899 nm comprises S_0_→S_39_ (83.90%) and S_0_→S_43_ (12.59%), while the broad weak peak at 1610 nm is almost exclusively due to S_0_→S_20_ (99.30%). A quantitative analysis reveals that 19.32% of the TbGe_12_^−^ absorption spectrum lies in the visible range, 20.35% in the near-infrared, and 61.08% in the mid-infrared region. For TbGe_16_^−^, its simulated UV–vis spectrum is shown in Figure 6. From it we can see that the spectrum exhibits a prominent absorption peak accompanied by two weaker peaks. The most intense absorption in the visible-light region occurs at 609 nm, primarily arising from the transitions S_0_→S_80_ (42.86%), S_0_→S_86_ (23.00%), and S_0_→S_69_ (20.93%). A second peak, located at the boundary between the visible and near-infrared regions, reaches its maximum at 788 nm, dominated by the transitions S_0_→S_31_ (66.49%) and S_0_→S_20_ (29.52%). In the near-infrared region, the adsorption peak at 956 nm is predominantly attributed to the S_0_→S_12_ (97.36%) transition. The UV–vis spectral coverage shows that the visible-light region accounts for 80.12% of the total spectral area, while the near-infrared region constitutes 19.85%. These results highlight the TbGe_16_^−^ and TbGe_12_^−^ clusters—highly symmetric structures with excellent stability—as a material with exceptional photoresponse properties. These findings suggest that the TbGe_16_^−^ and TbGe_12_^−^ clusters hold promise for applications in solar photothermal conversion, photoelectric conversion, and mid-infrared imaging technologies.

## 4. Conclusions

The geometrical evolution patterns and spectroscopic properties of Tb-doped germanium anionic nanoclusters (TbGe*_n_*^−^, *n* = 6–17) were systematically investigated using the ABCluster global search technique coupled with the mPW2PLYP double-hybrid density functional theory. The results demonstrate that the equilibrium geometries of the global minimum structures evolve toward the Tb-linked configuration, in which the Tb atom links two Ge sub-clusters starting from *n* = 10, while the Tb-centered Ge cage polyhedrons become energetically favored at *n* = 14. The photoelectron spectra of TbGe*_n_*^−^ (*n* = 6–17) nanoclusters were simulated, and vertical detachment energies were reported. The strong agreement between the theoretical and experimental photoelectron spectra for the TbGe_16_^−^ cluster validates the reliability of the predicted global minimum structures. Additionally, infrared and Raman spectra were computationally simulated. The stability analysis revealed that clusters with *n* = 12 and 16 exhibit not only superior thermal stability but also optimal chemical stability. A natural population analysis indicated that the total magnetic moments of TbGe*_n_*^−^ (*n* = 6–17) remain constant at 6 μ*_B_*, primarily originating from the Tb atom. Although 4*f* electrons participate in bonding, their antiparallel spin configuration persists, resulting in the magnetic moments of the TbGe*_n_*^−^ (*n* = 6–17) nanoclusters matching that of an isolated Tb atom. The UV–Vis spectra of the TbGe_12_⁻ and TbGe_16_^−^ clusters not only overlap with the solar visible spectrum but also exhibit strong absorption peaks in the near-infrared and mid-infrared regions, demonstrating exceptional light-harvesting capabilities. With their remarkable stability and tunable photothermal properties, TbGe_12_^−^ and TbGe_16_^−^ clusters can serve as ideal building blocks for developing novel functional nanomaterials, showing promising applications in solar photothermal conversion, photoelectric conversion, and infrared imaging technologies through their distinct one- and three-dimensional architectures, respectively.

## Figures and Tables

**Table 1:** The NPA valence electron configuration, charge of the Tb atom, each valence orbital, and total magnetic moments of the GM structure for TbGe*_n_*^−^ (*n* = 6–17) calculated with the mPW2PLYP scheme.

Clusters	Charge (a.u.)	Electron Configuration	Magnetic Moment of Tb Atom						Molecule (μ*_B_*)
			6*s*	4*f*	5*d*	6*p*	7*s*	Total	
TbGe_6_^−^	0.07	[core]6*s*^0.82^4*f*^7.36^5*d*^2.21^6*p*^0.31^7*s*^0.24^	−0.50	6.59	0.28	0.01	−0.04	6.34	6
TbGe_7_^−^	0.17	[core]6*s*^0.75^4*f*^7.36^5*d*^2.17^6*p*^0.29^7*s*^0.25^	−0.52	6.44	0.26	0.01	−0.03	6.16	6
TbGe_8_^−^	0.19	[core]6*s*^0.78^4*f*^7.38^5*d*^2.08^6*p*^0.36^7*s*^0.21^	−0.54	6.58	0.26	0.01	−0.03	6.28	6
TbGe_9_^−^	0.39	[core]6*s*^1.28^4*f*^7.00^5*d*^1.73^6*p*^0.43^7*s*^0.17^	0.46	6.96	−0.50	−0.27	−0.04	6.61	6
TbGe_10_^−^	0.32	[core]6*s*^1.01^4*f*^7.01^5*d*^1.96^6*p*^0.58^7*s*^0.11^	−0.74	6.95	0.04	0.02	0.01	6.28	6
TbGe_11_^−^	0.12	[core]6*s*^0.71^4*f*^7.36^5*d*^1.88^6*p*^0.68^7*s*^0.25^	−0.53	6.60	0.15	0.02	−0.07	6.17	6
TbGe_12_^−^	−0.27	[core]6*s*^0.71^4*f*^7.35^5*d*^2.15^6*p*^0.81^7*s*^0.24^	−0.54	6.61	0.15	0.01	−0.06	6.17	6
TbGe_13_^−^	0.10	[core]6*s*^0.73^4*f*^7.34^5*d*^2.35^6*p*^0.39^7*s*^0.13^	−0.57	6.62	0.26	−0.01	−0.01	6.29	6
TbGe_14_^−^	−2.94	[core]6*s*^0.70^4*f*^7.35^5*d*^4.17^6*p*^1.37^7*s*^0.33^	−0.71	6.94	0.15	0.02	−0.01	6.16	6
TbGe_15_^−^	−3.94	[core]6*s*^0.82^4*f*^6.99^5*d*^5.06^6*p*^1.74^7*s*^0.27^	−0.60	6.95	0.26	0.02	−0.05	6.58	6
TbGe_16_^−^	−4.76	[core]6*s*^0.66^4*f*^7.35^5*d*^5.28^6*p*^2.00^7*s*^0.41^	−0.43	6.61	0.20	0.01	−0.18	6.21	6
TbGe_17_^−^	−3.93	[core]6*s*^0.67^4*f*^7.35^5*d*^4.81^6*p*^1.68^7*s*^0.37^	−0.46	6.61	0.19	0.01	−0.15	6.20	6

## Data Availability

Data will be made available on request.
